# Environment Controls LEE Regulation in Enteropathogenic *Escherichia coli*

**DOI:** 10.3389/fmicb.2018.01694

**Published:** 2018-07-27

**Authors:** Amy Platenkamp, Jay L. Mellies

**Affiliations:** Department of Biology, Reed College, Portland, OR, United States

**Keywords:** EPEC, LEE, environment, virulence, sRNA, metabolism, envelope stress

## Abstract

Enteropathogenic *Escherichia coli* (EPEC) is a significant cause of infant morbidity and mortality in developing regions of the world. Horizontally acquired genetic elements encode virulence structures, effectors, and regulators that promote bacterial colonization and disease. One such genetic element, the locus of enterocyte effacement (LEE), encodes the type three secretion system (T3SS) which acts as a bridge between bacterial and host cells to pass effector molecules that exert changes on the host. Due to its importance in EPEC virulence, regulation of the LEE has been of high priority and its investigation has elucidated many virulence regulators, including master regulator of the LEE Ler, H-NS, other nucleoid-associated proteins, GrlA, and PerC. Media type, environmental signals, sRNA signaling, metabolic processes, and stress responses have profound, strain-specific effects on regulators and LEE expression, and thus T3SS formation. Here we review virulence gene regulation in EPEC, which includes approaches for lessening disease by exploiting the elucidated regulatory pathways.

## Introduction

Diarrheal disease is a leading cause of infant death worldwide, according to the World Health Organization. Enteropathogenic *Escherichia coli* (EPEC) is a major player in diarrheal disease—a study in Nairobi, Kenya of 207 clinical isolates from children under five with diarrhea found that the majority (19.3%) of isolates were EPEC (Makobe et al., 2012). Additionally, a Global Enteric Multicenter Study of children in sub-Saharan Africa and south Asia found that EPEC is associated with increased risk of death in children ages 0–11 months (Kotloff et al., 2013). EPEC shares many genetic and phenotypic similarities with enterohemorrhagic *E. coli* (EHEC), a zoonotic pathogen that infects children and adults, with a major reservoir being livestock (Hartland and Leong, 2013). EPEC and EHEC have been implicated in numerous acute diarrheal outbreaks worldwide (Majowicz et al., 2014; Hu and Torres, 2015). EPEC’s burden on children in developing countries and its relatedness to an infectious strain with a broader host range, namely EHEC, has incited bacteriologists to extensively study its disease mechanism in a collective, ongoing investigation.

Enteropathogenic *Escherichia coli* belongs to an over-arching group of infectious *E. coli*, referred to as attaching and effacing *E. coli* (AEEC). Attaching and effacing (A/E) pathogens are referred to as such because they intimately adhere to the host cell surface, efface brush border microvilli, and form characteristic actin-rich pedestals. Strains in this group are defined by the presence of a 35–43 kb horizontally acquired pathogenicity island named the locus of enterocyte effacement (LEE), which has inserted into various *E. coli* strains in many independent instances (Elliott et al., 1998; Hazen et al., 2013). The EPEC strain E2348/69 LEE contains 41 open reading frames with an average GC-content of 38.3% compared to the genome average of 50.6% (Elliott et al., 1998; Iguchi et al., 2009). Operons *LEE1, LEE2*, and *LEE3* encode the type three secretion system (T3SS) inner and outer membrane components including SepD and SepQ, as well as the outer membrane porin EscC and the ATPase EscN (Elliott et al., 1998). *LEE4* encodes the filament structure protein EspA and translocator proteins EspD and EspB along with other structural proteins and SepL (Elliott et al., 1998). *LEE5* encodes intimin and translocated intimin receptor (Tir), which facilitate intimate attachment of EPEC cells to the epithelium, as well as the Tir chaperone CesT (Sánchez-SanMartín et al., 2001). Other genes of the LEE include *espG, grlRA, cesF, map*, and *escD* (Iguchi et al., 2009). The *LEE1* operon encodes a master regulator of the LEE PAI named the LEE-encoded regulator (Ler) (Mellies et al., 1999; Elliott et al., 2000; Sperandio et al., 2000; Bustamante et al., 2001; Sánchez-SanMartín et al., 2001; Haack et al., 2003; Li et al., 2004; Bingle et al., 2014). Cloning the EPEC LEE pathogenicity island from the archetypal E2348/69 strain into an *E. coli* K-12 strain confers the A/E phenotype on cultured epithelial cells (McDaniel and Kaper, 1997). Mutations in the *eae* gene encoding intimin show decreased virulence- diminished colonic hyperplasia and CFU per gram of tissue using the A/E pathogen *Citrobacter rodentium* in a mouse model of infection (Reece et al., 2002), and Tir is essential for virulence in mice (Deng et al., 2003). Deletion of *ler* results in loss of LEE expression, effector secretion, pedestal formation, and virulence in the mouse model (Deng et al., 2004). Use of the A/E pathogen *C. rodentium*, as an infection model of LEE-associated virulence in mice has been reviewed previously (Mundy et al., 2005; Yang et al., 2010; Collins et al., 2014). Similarly, in rabbit EPEC (rEPEC), a *ler* deletion mutant is well tolerated by the animals whereas the wt parent strain causes severe diarrhea (Zhu et al., 2006). The LEE is the common genetic element of AEEC.

Though EPEC acquired the LEE, they are distinct from other AEEC in certain ways. EPEC lack the EHEC-containing Shiga-toxin (stx) that results in painful, bloody stool and can cause the complication known as hemolytic uremic syndrome in about 15% of cases (Tarr et al., 2005). Typical EPEC (tEPEC) contain the LEE and 14 genes that encode the bundle-forming pilus (BFP), which is most often encoded on the 70- to 95-kb (without or with *tra* transfer genes, respectively) *E. coli* adherence factor plasmid (pEAF). Atypical EPEC (aEPEC) contain the LEE and, and by definition, lack *bfp* (and the pEAF in most cases) (Brinkley et al., 2006; Hazen et al., 2013, 2015b). The pEAF additionally contains the plasmid encoded regulators, *perABC*, also termed *bfpTVW* (Tobe et al., 1996; Okeke et al., 2001; Brinkley et al., 2006). EPEC is parsed into six lineages, of which two are typical EPEC and the remaining are atypical (Hazen et al., 2013). Infection with typical EPEC can be asymptomatic to causing severe, acute watery diarrhea (Ahmed et al., 2014; Hu and Torres, 2015), which can become lethal. Some data may indicate that aEPEC is less virulent than typical strains containing the pEAF plasmid and the bundle-forming pilus (Levine et al., 1985), but clinical data indicate that aEPEC cause prolonged diarrhea. In a study by Nguyen et al. (2006), the mean number of days with aEPEC diarrhea was 12.1 (Nguyen et al., 2006). In many parts of the world, aEPEC is becoming more prevalent than tEPEC (Hu and Torres, 2015).

Enteropathogenic *Escherichia coli* spreads via the ‘fecal-oral route,’ which necessitates its ability to withstand not only the environment exterior to the host, but also the acidic environment of the stomach and the viscous mucous layer atop the epithelium. In colonization of the distal small intestine, tEPEC uses flagella to navigate the lumen and mucous layer, and utilizes both flagella and BFP to attach to the apical epithelial lining and accumulate together in micro-colonies of five to 200 bacteria, forming the ‘localized adherence’ (LA) phenotype (Tobe and Sasakawa, 2001; Giron et al., 2002; Cleary et al., 2004). Although none are as well characterized as the LA phenotype, aEPEC strains attach to the epithelial lining by various mechanisms such as ‘LA-like’ attachment, diffuse adherence, or aggregative adherence (Scaletsky et al., 2009, 2010).

Upon attachment, EPEC forms the T3SS that provides a bridge between the bacterial and the host cell cytoplasms, through which effector molecules pass from bacteria to host. Upon attachment of the T3SS to the host cell, the Tir protein is translocated into the cytoplasm where it then embeds into the membrane and is phosphorylated by host cell tyrosine kinases (Kenny et al., 1997b; Kenny, 1999). Actin recruitment occurs, forming pedestals on which the bacterium sits, with tight attachment resulting when the bacterial outer membrane protein intimin binds to Tir (reviewed in Kaper et al., 2004; Frankel and Phillips, 2008; Wong et al., 2011; Pearson et al., 2016). Strain E2348/69 has 7 LEE-encoded translocated effectors (Tir, Map, EspF, EspG, EspH, SepZ, and EspB) and fourteen non-LEE encoded (*nle*) effectors on prophages and integrative elements (Garmendia et al., 2005; Iguchi et al., 2009). Effector molecules work to further stabilize bacterial attachment to the apical enterocyte membrane, disrupt tight junctions between enterocytes, efface brush border microvilli, and cause actin polymerization and accumulation beneath the adherent bacterium, creating the characteristic cup-like pedestal in what is termed the attaching and effacing (A/E) lesion (Knutton et al., 1993). Infection by EPEC causes persistent secretory diarrhea, low fever, anorexia, and can cause death within a few days (Arenas-Hernández et al., 2012). Secretory diarrhea is characterized by increased chloride secretion, decreased sodium absorption, and increased mucosal permeability (Arenas-Hernández et al., 2012). Effector molecule deployment through the LEE-encoded T3SS is thought to allow EPEC to evade the host intestinal immune system by inhibiting phagocytosis, protein secretion, inflammation, and apoptotic cell death, which is reviewed in depth elsewhere (Santos and Finlay, 2015; Pearson et al., 2016).

Connections between environmental signals and regulation of the LEE and other aspects of virulence have shown that many stimuli can have profound effects on EPEC virulence. Specific, tightly regulated control of virulence gene expression ensure that EPEC deploys colonization tactics once in its ideal niche. Connolly et al. (2015) reviewed the effects of environment on EHEC LEE expression, thus this review aims to summarize the environmental effects on EPEC virulence gene regulation. Here, we discuss the mechanisms of the key regulators PerC, GrlA, and Ler, further regulation of the LEE, and manners in which LEE regulation and other virulence mechanisms are controlled in response to environmental stimuli.

## Lee Regulation

**FIGURE 1:**
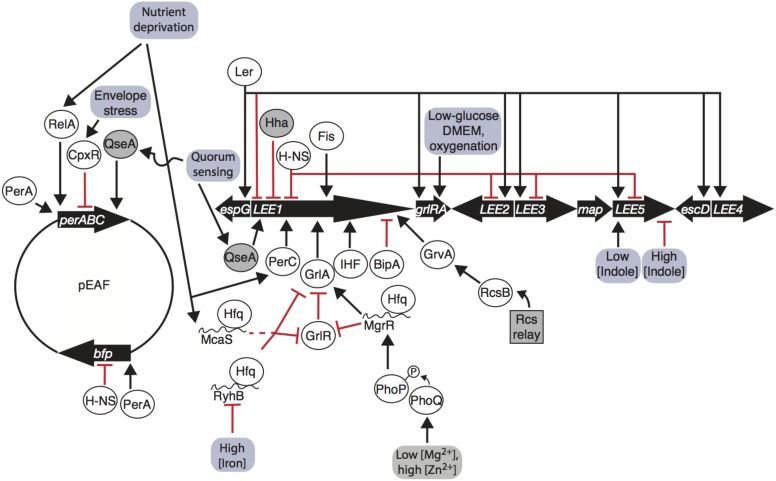
Enteropathogenic *Escherichia coli* (EPEC) LEE and pEAF gene regulation is influenced by environmental inputs. The *LEE1* operon encodes Ler, which promotes transcription of all operons and auto-represses its own promoter. *LEE1* is regulated by a number of nucleoid associated proteins (Ler, H-NS, Hha, Fis, and IHF), pEAF-encoded regulator PerC, LEE-encoded GrlA/GrlR, quorum-sensing factor QseA, BipA, and GrvA. RelA, CpxR, and QseA regulate the *per* operon on the pEAF, PerA promotes *bfp* transcription and auto-activates *per*. Environmental signals are transmitted through phosphorelay systems, sRNAs, stringent responses, quorum-sensing responses, and envelope stress responses to affect transcription of the LEE and pEAF. Arrows indicate transcription/translation promotion, blunt arrows indicate transcription/translation repression; proteins are in ovals; sRNAs are indicated by curvy lines; genetic elements are indicated by thick black arrows; environmental inputs are in red boxes; dashed line indicates indirect regulation.

Regulation of the LEE is controlled by many factors, including a range of nucleoid-associated proteins (**Table 1**). Master regulator Ler is encoded on the first open reading frame of *LEE1*, and is a transcriptional activator of *LEE2, LEE3, LEE4*, and *LEE5*, as well as extra-LEE genes including *nleI/G* to *nleF*, *espC*, and an *espG* homolog (Mellies et al., 1999; Elliott et al., 2000; Sperandio et al., 2000; Bustamante et al., 2001; Sánchez-SanMartín et al., 2001; Haack et al., 2003; Li et al., 2004; Bingle et al., 2014) (**Figure 1**). Ler represses expression of its own operon (Bhat et al., 2014). The importance of Ler in greater regulation of the LEE has driven investigation of the regulators and environmental signals affecting *LEE1*. The *LEE1* operon has two promoters, *LEE1* P1A and P1B, of which P1B is dominant in transcripts and in translated products (Jeong et al., 2012). Transcription off of the P1B promoter, but not the P1A promoter, is dependent on integration host factor (IHF), which binds to the promoter and induces expression (Friedberg et al., 1999; Jeong et al., 2012). Another DNA-binding protein, factor for inversion stimulation (Fis), increases transcription of the *LEE1* by bending DNA (Pan et al., 1996; Goldberg et al., 2001). Hha binds to the *LEE1* promoter and represses transcription in EHEC (Sharma and Zuerner, 2004). Non-nucleoid associated factors that positively regulate *LEE1* include BipA and the quorum sensing regulator QseA (**Figure 1**). The ribosome-binding GTPase BipA decreases transcription of *ler* in a Per, IHF, and H-NS independent manner (Grant et al., 2003). In EHEC, QseA directly binds the *LEE1* promoter and activates transcription (Sharp and Sperandio, 2007). The effect of these factors on transcription of the LEE, particularly *LEE1* encoding Ler, is important for virulence (Deng et al., 2004; Zhu et al., 2006) (**Figure 1**).

**Table 1:** Summary of regulatory factors, their roles, and environmental signals that control them.

Factor	Role	Environmental signals	Reference
BipA	Ribosome-binding GTPase, decreases *LEE1* transcription		Grant et al., 2003
CpxRA	CpxA phosphorylates transcriptional regulator CpxR	Alkaline pH, antibiotics, ethanol, growth, high osmolarity, indole, n-butanol, adherence, copper	Danese and Silhavy, 1998; Otto and Silhavy, 2002; Raffa and Raivio, 2002; DiGiuseppe and Silhavy, 2003; Jubelin et al., 2005; Yamamoto and Ishihama, 2006; Wolfe et al., 2008; Bury-Moné et al., 2009; Rutherford et al., 2010; Kashyap et al., 2011
CsrA	Global metabolism regulator, represses *grlA*, stabilizes *sepL* and *tnaA* mRNAs, binds CesT		Bhatt et al., 2009; Katsowich et al., 2017; Ye et al., 2018
CsrB	sRNA, sequesters CsrA		Yakhnin et al., 2017
CsrC	sRNA, sequesters CsrA		Yakhnin et al., 2017
DegP	Degrades misfolded periplasmic envelope proteins, cleaves E-cadherin and BFP	In response to σ^E^ and Cpx activation	Hyland et al., 2008; Humphries et al., 2010; MacRitchie et al., 2012; Abfalter et al., 2016
DsbA	Disulfide oxidase, stabilizes bundilin	In response to Cpx activation	Zhang and Donnenberg, 1996; Heras et al., 2009; Vogt et al., 2010
EscP	SepL-associated mediation of effector secretion	Calcium	Shaulov et al., 2017
EscV	Interacts with SepL to mediate translocator and effector secretion		Portaliou et al., 2017
Fis	Nucleoid-associated activator of *LEE1*	Depressed by ppGpp overproduction	Goldberg et al., 2001; Brandi et al., 2016
GrlA	Activates *LEE1* transcription	Low-glucose DMEM (vs. LB), oxygenation	Padavannil et al., 2013
GrlR	Binds and represses GrlA	Same as GrlA	Barba et al., 2005; Jimenez et al., 2010; Padavannil et al., 2013
H-NS	Nucleoid-associated repressor of LEE		Mellies et al., 2011; Leh et al., 2017
Hha	Nucleoid-associated repressor of *LEE1* in EHEC		Sharma and Zuerner, 2004
IHF	Nucleoid-associated activator of *LEE1*, by P1B promoter		Friedberg et al., 1999; Jeong et al., 2012
Ler	Activator of LEE PAI, repressor of *per*		Mellies et al., 1999; Bingle et al., 2014
McaS	sRNA, sequesters CsrA		Bhatt et al., 2017
MdtABC	Multi-drug efflux pump	Same as BaeSR	Lee et al., 2005
MgrR	sRNA, stabilizes GrlA transcripts	Low magnesium concentrations	Bhatt et al., 2017
NlpE	Activates CxpAR	Abiotic surface adherence	Grabowicz and Silhavy, 2017
PerA	Auto-inducer of *per* operon, induces *bpf* transcription	ppGpp production	Spira et al., 2014
PerB	Implicated in BFP activation	Same as PerA	Gomez-Duarte and Kaper, 1995; Tobe et al., 1996
PerC	Positive regulator of *LEE1*	Same as PerA	Porter et al., 2005; Bustamante et al., 2011
PpiA	Facilitates formation of T3SS and flagella	In response to Cpx activation	Justice et al., 2005; MacRitchie et al., 2012
RelA	ppGpp synthetase	Amino acid starvation	Wendrich et al., 2002
RpoE (σ^E^)	Alternative sigma factor	Heat, ethanol, n-butanol, zinc	Erickson and Gross, 1989; Yamamoto and Ishihama, 2005; Rutherford et al., 2010; Yitzhaki et al., 2012
RyhR	sRNA, represses GrlRA translation	Repressed by high iron concentrations	Bhatt et al., 2017
SepD	Mediates SepL and EscV to control translocator and effector secretion		Gaytán et al., 2017; Portaliou et al., 2017
SepL	Modulates timing of effector secretion	Calcium	Shaulov et al., 2017
SpoT	ppGpp synthetase/hydrolase	Synthesizes ppGpp in response to carbon starvation, fatty acid starvation, iron limitation	Edwards et al., 2011
TnaA	Cleaves tryptophan into indole, pyruvate, and ammonia	Indirectly promoted by CsrA	Bhatt et al., 2011
TnaB	Tryptophan permease		Bhatt et al., 2011
TnaC	*Cis-*acting regulator		Bhatt et al., 2011
YjfN	Activator and substrate of DegP		Kim et al., 2018

### Ler and H-Ns

The mechanisms of action of Ler and H-NS at LEE operons have been investigated extensively. Data indicate Ler increases transcription by displacing the silencer H-NS and other nucleoid-associated proteins, which permits transcription (Friedberg et al., 1999; Sánchez-SanMartín et al., 2001; Mellies et al., 2011). Recently, Shin (2017) demonstrated using *lacZ* reporter gene fusions that Ler only activates the *LEE5* promoter in the presence of H-NS. This result was recapitulated by *in vitro* transcription—the relative number of transcripts off of the *LEE5* promoter were the same with and without Ler in the absence of H-NS (Shin, 2017). However, there is some evidence that Ler regulates *LEE5* in both H-NS-dependent and H-NS-independent manners (Sánchez-SanMartín et al., 2001; Umanski et al., 2002). Predictably, the dissociation constants of Ler to P*_LEE5_* (*K*_d_ = 4.59 × 10^-8^) and that of H-NS to P*_LEE5_* (*K*_d_ = 1.43 × 10^-7^) by surface plasmon resonance indicate that Ler has a greater binding affinity to the promoter than H-NS (Shin, 2017). The C-terminal regions of the proteins are the most conserved (Sperandio et al., 2000). Mutations in this conserved region abolishes Ler and H-NS binding to DNA (Yerushalmi et al., 2008; Sette et al., 2009).

The structures of Ler and H-NS, and how the proteins interact with DNA have also been investigated. A Ler 70–116 peptide binds to the minor groove of AT-rich DNA in the *LEE2/LEE3* regulatory region, where Ler was shown to bind by DNA footprinting experiments (Sperandio et al., 2000; Cordeiro et al., 2011). For both proteins, A Q/RGR loop motif in the C-terminus mediates binding (Gordon et al., 2011). H-NS forms head-to-head, tail-to-tail multimers, bridging DNA and forming plectonemic structures (Arold et al., 2010), while Ler wraps DNA, forming toroids (Mellies et al., 2011). At *LEE5*, Ler binds over an extended region between -190 and -70 (Haack et al., 2003), while at most promoters H-NS binds upstream and downstream of the transcriptional start site. +1. In addition, data indicate that H-NS binds cooperatively, while Ler binds non-cooperatively to DNA (Winardhi et al., 2014). Ler has a shorter N-terminal coiled-coil domain than H-NS, containing only two α-helices and is “missing” the H-NS residues 59–68, which in H-NS are key in stabilizing dimerization (Arold et al., 2010), suggesting that Ler does not oligomerize in the same fashion as H-NS. Thus, Ler and H-NS recognize similar or identical motifs, but the manner in which they oligomerize and alter DNA structure differs (Mellies et al., 2011; Leh et al., 2017). Investigating the signaling affecting *LEE1* transcription, and the molecular mechanisms of Ler-mediated control of LEE expression is paramount for understanding EPEC virulence.

### Global Regulator of Ler (GrlA)

The Global regulator of Ler (GrlA) activates transcription of *LEE1* in EPEC (**Figure 1**) and EHEC, controls transcription of a hemolysin and its secretion machinery encoded by *ehxCABD* and the flagellar regulator genes *flhDC* in EHEC (Padavannil et al., 2013). *grlA* is transcribed with its repressor *grlR* in an operon within the LEE PAI (Barba et al., 2005). GrlA activates Ler independently of PerC and IHF, and Ler increases GrlRA expression in a positive feedback loop (Barba et al., 2005). GrlA binds to the *LEE1* promoter between -54 and the transcriptional start site (Padavannil et al., 2013). GrlR binds to the GrlA, outcompeting the *LEE1*, *ehxCABD*, and *flhDC* promoters and diminishing transcription (Jimenez et al., 2010; Padavannil et al., 2013). GrlR dimerizes and interacts with the HTH domain and C-terminus of a GrlA monomer, blocking the DNA-binding region GrlA (Padavannil et al., 2013). Thus, GrlA binds to DNA to positively regulate hemolysis, motility, and the LEE through master virulence regulator *ler*, and GrlA and the plasmid-encoded regulator *perC* activate the *LEE1* operon in parallel.

### The *per* Operon

Plasmid-encoded genes have been implicated in aiding bacteria to survive in specific local environments (Eberhard, 1989). The pEAF encodes two major operons, *bfp* and *per*, that advantage tEPEC in colonizing the human small intestine by production of type IV pili and promoting transcription of the *LEE*, respectively (**Figure 1**). Expressed from the *perABC* operon, PerA induces *bfp* transcription and auto-activates *per* (Gomez-Duarte and Kaper, 1995; Porter et al., 2004). PerA is part of the AraC/XylS family of transcriptional regulators, a group of over 1,500 proteins known to regulate adaptation to environmental changes and stress responses, carbon and nitrogen metabolism, and virulence (Ibarra et al., 2013). Regulators of this family have a DNA-binding domain in the C-terminus and two non-conserved HTH structures in the N-terminus, typically for dimerization and effector molecule-binding (Ibarra et al., 2013). The C- and N-termini in PerA contain key residues that allow PerA to bind to DNA, likely as a monomer (Porter et al., 2004; Ibarra et al., 2013). PerA binds to an AT-rich 29-bp sequence upstream of the -35 positions of the transcriptional start sites of *bfp* and *per* and increases transcription of both operons (Porter et al., 2004; Ibarra et al., 2013). The function of PerB is largely uncharacterized, but it is thought that PerB aids in activating *bfp* (Gomez-Duarte and Kaper, 1995; Tobe et al., 1996). Recent findings indicate that PerA and PerB are involved in greater control of the EPEC transcriptome.

An initial observation of smaller and larger colony morphologies of EPEC when cultured in low-glucose DMEM and then plated on agar led to the finding that bimodal *per* expression is correlated with bimodal colony morphologies controlled by PerA and PerB (Leh et al., 2017; Ronin et al., 2017). Small colonies are associated with high *per* expression and large with weak *per* expression (Leh et al., 2017; Ronin et al., 2017). It is argued that *per* expression dictates distinct transcriptomes among subpopulations, which advantages EPEC in unpredictable milieus (Leh et al., 2017; Ronin et al., 2017). Remarkably, bimodal colony morphology can be induced in *E. coli* K-12 strain MG1655 by expression of *perAB*, indicating that the role of *per* on colony morphology bimodality is not solely dependent on its transcription of the LEE PAI (Ronin et al., 2017). The small colonies showed increased aggregation and microcolony formation on cultured epithelial cells, but it is unknown how these different morphologies might affect virulence in an *in vivo* infection model, or contribute to disease (Ronin et al., 2017). However, it is becoming increasingly evident that *per* has an important role in allocation of transcriptional resources in response to nutrient availability in addition to encoding a virulence gene activator.

The third *per-*encoded protein, PerC activates the *LEE1* promoter which encodes Ler, thereby stimulating virulence gene expression encoded in the LEE PAI (Mellies et al., 1999; Bustamante et al., 2011). Along with stimulating virulence gene expression, PerC regulates many genes. RNA-sequencing revealed that PerC regulates, either directly or indirectly, transcription of the *fim* operon, various metabolism genes, ribosomal RNAs, tRNAs, sRNAs, and others (Mellies et al., 2017). PerC’s mechanism of action has remained largely elusive, but some observations are worth examining. It is known that PerC requires DNA from positions -54 to +216 in relation to the start site of *LEE1* to activate transcription (Bustamante et al., 2011). It has been hypothesized that PerC homologs (Pch) in EHEC bind to DNA and de-repress transcriptional activity by dislodging H-NS in a manner similar to Ler because of the following findings: PchA binds preferentially to AT-rich DNA or DNA complexes, Pch reduces the amount of H-NS bound to DNA, Pch and Ler preferentially bind in H-NS binding regions, and Pch is dependent on Hha and H-NS for activation of *LEE1* in EHEC (Fukui et al., 2016). In contradiction, Porter et al. (2005) was unable to detect EPEC *LEE1* promoter DNA-PerC complexes *in vitro* by electrophoretic mobility shift assay to a protein concentration of 1 μM, and thus concluded that PerC either does not bind to DNA, or only binds in the presence of other factors (Porter et al., 2005). Additionally, PerC in EPEC does not require H-NS to activate transcription—an IHF and H-NS double mutant maintains PerC-dependent activation of *LEE1*, indicating that PerC activation is not IHF or H-NS dependent, rather the two nucleoid-associated proteins act antagonistically at the *LEE1* promoter (Porter et al., 2005). Taken together, it is undetermined whether PerC binds to DNA in the presence of other proteins or acts by another mechanism in which it does not bind to DNA. Thus, PerC regulates beyond *LEE1* activation by an unknown mechanism.

Per and Ler regulation are intimately linked (**Figure 1**). A microarray study revealed that Ler represses *per* expression roughly threefold, indicating a negative feedback loop (Bingle et al., 2014). The intertwined regulation of these important players has implications in interpretation of their observed regulons. For instance, an increase in *bfp* transcripts in a *ler* mutant is likely due to decreased repression of *perA*, a known positive regulator of *bfp* (Bustamante et al., 2011; Bingle et al., 2014; Mellies et al., 2017). Conversely, the *fim* operon was found to be increased in expression in both *perC*- and *ler-*deletion mutants. Down-regulation of *fim* by these regulators could possibly be explained by decreased *fimE* recombinase transcription, inverting the *fimS* element to the OFF orientation due to Ler in both cases (Bingle et al., 2014; Mellies et al., 2017). The negative feedback loop between *per* and Ler dictates controlled regulation of the LEE, which is also coordinated by environmental signals.

## Environmental Signals

Nutritional stress has a profound effect on virulence gene expression. Initially, it was observed that low-glucose DMEM, which has 5.5 mM D-glucose, or 0.1% (while high-glucose DMEM has 25 mM D-glucose, or 0.45%), provides increased expression of virulence genes compared to LB when grown at 37°C to an OD_600_ of 0.2 to 0.5 (Puente et al., 1996; Rosenshine et al., 1996; Leverton and Kaper, 2005; Bustamante et al., 2011; Hazen et al., 2015a). *per*, *bfpA*, and *grlA* transcription are all increased more than 4 log_2_ fold compared to LB at an OD_600_ of 0.5 in strain E2348/69, and similarly *ler* is expressed over 2 log_2_ fold in this strain (Hazen et al., 2015a). This pattern of regulation is not upheld in all tEPEC strains tested by Hazen et al. (2015a)—in fact, *perC* has decreased expression in tEPEC strain C581-05 under these conditions, and *ler* and *grlA* expression was unchanged (Hazen et al., 2015a). Growth conditions further dictate LEE expression—in a Δ*perC* mutant, *ler* transcription is 5.5-fold less than WT in DMEM with 0.45% glucose and grown statically with 5% CO_2_, but unchanged in shaking conditions without added CO_2_ (Bustamante et al., 2011). This regulation has downstream effects, as effector secretion of EspA, EspB, EspC, and EspD is decreased under static conditions, but not shaken conditions (Bustamante et al., 2011). Additionally, butyrate, which is abundant in the colon where EHEC colonizes, has been shown to increase *pchA* expression in EHEC (Nakanishi et al., 2009). Growth conditions affect GrlA-mediated control of the LEE as well. In *grlA* mutant, *ler* transcription was decreased fivefold when shaken in DMEM with 0.45% glucose and without CO_2_, but transcription was not decreased in static growth conditions with CO_2_ (Barba et al., 2005; Bustamante et al., 2011). Importantly, environmental signals have a significant influence on regulators, and ultimately affect timing of virulence gene expression.

The *per* operon is implicated in affecting timing of virulence expression. *per* is transcribed in response to environmental stimuli and in turn modulates expression of the *LEE* PAI and other genes that are predicted to advantage tEPEC in its ileal niche (Nakanishi et al., 2006; Spira et al., 2014; Leh et al., 2017; Mellies et al., 2017; Ronin et al., 2017). *per* gene expression occurs maximally in early exponential growth and has been implicated in the temporal control of colonization (Bustamante et al., 2011; Bueris et al., 2015). Surprisingly, aEPEC can be induced to colonize earlier when *per* is introduced in *trans* (Bueris et al., 2015). These data indicate the larger role in timing of virulence gene expression in response to beneficial environments for colonization.

These initial observations of the effect of media type on virulence gene transcription led to the finding that it is the transition from nutrient-dense LB to low-glucose DMEM that induces virulence gene expression in EHEC (Nakanishi et al., 2006). This transition induces the stringent response, in which *relA* and *spoT* encoding synthetases of guanosine (penta-) tetra-phosphate (herein collectively referred to as ppGpp), and DksA are necessary for the growth-phase dependent increase in *LEE1* expression in EHEC (Nakanishi et al., 2006). RelA overexpression increases EspB and Tir secretion in the presence of functional *pchA* and *ler* genes in EHEC (Nakanishi et al., 2006). Intriguingly, in EPEC, RelA induces *per* expression, is necessary for *bfp* expression at WT levels, and plays a role in adherence to HEp-2 cells (Spira et al., 2014). Additionally, ppGpp over production depresses Fis expression, and to a lesser extent that of H-NS (Brandi et al., 2016). Fis also increases transcription of many genes involved in primary metabolism, such as tRNAs and rRNAs, likely because those are the genes that are being expressed in the early exponential growth phase when Fis is expressed (Ross et al., 1990; González-Gil et al., 1996; Goldberg et al., 2001). Nutrient deprivation is sensed and relayed through the stringent response to control *per* and virulence gene expression. Beyond growth conditions, other virulence determinants have been linked to environmental signal sensing, including calcium-mediated secretion system assembly, sRNA global regulation and metabolism, and envelope stress induced mediation of virulence factors.

## Type Three Secretion

### Calcium-Mediated Secretion

Calcium-deplete conditions have been observed to reduce secretion of the earlier translocators and cause “hypersecretion” of the later secreted effectors through the T3SS, likely by signaling that is transmitted through the gatekeeper protein SepL and/or through the “ruler” protein EscP (Kenny et al., 1997a; Ide et al., 2003; Deng et al., 2004; Gaytán et al., 2017; Shaulov et al., 2017). The membrane-associated translocator receptor SepL has been implicated in modulating the switch from early substrates to translocators and from translocators to effectors by interacting with different proteins in various mechanisms (Portaliou et al., 2017; Shaulov et al., 2017). It’s suggested that effector secretion is blocked by an interaction of SepL and EscP, and in calcium-deplete conditions, SepL and EscP dissociate and effectors are secreted (Shaulov et al., 2017). Shaulov et al. (2017) suggest that the calcium-rich extracellular milieu leaks in to the base of the basal body during T3SS formation, and upon completion of the T3SS and closed connection of bacterial and host cells, the calcium concentration drops to intracellular levels, causing a dissociation of SepL and EscP (Monjarás Feria et al., 2012; Shaulov et al., 2017). Many studies have observed calcium-dependent altered secretion, and this is one possible mechanism for these observations.

Another mechanism provides a calcium-independent explanation for control of secretion by SepL. A stepwise theory in which early substrates, then translocators, and finally effectors are secreted necessitates that SepL, SepD, and EscV interact in a single mechanism to control both translocator and effector secretion (Portaliou et al., 2017). SepL interacts with SepD in a calcium-independent manner, in which SepD mediates the crosstalk between SepL and the major T3SS component EscV, activating SepL and permitting translocator targeted secretion while blocking effector secretion (Gaytán et al., 2017; Portaliou et al., 2017). SepD releases, uncoupling EscV and SepL crosstalk and permitting both translocator and effector secretion (Portaliou et al., 2017). Then, the translocator receptor SepL releases, freeing up space for effectors to bind EscV and to be secreted (Portaliou et al., 2017). By these two studies, it’s suggested that SepL is involved in controlling the timing of secretion in parallel, calcium-dependent and -independent mechanisms.

### Disruption of Host Homeostasis

Enteropathogenic *Escherichia coli* infection, by T3SS-mediated mechanisms, disrupts a number of host metabolic and physiologic processes. Live EPEC with a functional T3SS, but not non-pathogenic *E. coli*, dead EPEC, nor EPEC supernatant, decreases vitamin B1 (thiamin) uptake of Caco-2 cells by affecting the thiamin uptake receptors hTHTR-1 and hTHTR-2 (Ashokkumar et al., 2009). Effectors *espF* and *espH* at least partially depress thiamin uptake (Ashokkumar et al., 2009). EPEC, in a T3SS-dependent manner, induces activity of Na^+^/H^+^ transporters NHE1 and NHE2, thus increasing Na^+^ cation uptake, disrupting electrolyte homeostasis (Hecht et al., 2004). In fact, EPEC infection causes an increase in epithelial short circuit current that is partially dependent on an induction of chloride secretion (Collington et al., 1998). EPEC infection causes an increase in protein tyrosine phosphatase (PTPase), specifically by activation of Src-homology-2 domain containing PTPase, which decreases tyrosine phosphorylation of the serotonin transporter, decreasing transporter function and thus deregulation of serotonin uptake (Singhal et al., 2017). The EPEC T3SS is necessary for many instances of host homeostasis disruption.

## sRNA Signaling and Bacterial Metabolism

### Small RNAs and Their Chaperones

Small RNAs (sRNAs) are regulators of many metabolic processes, motility, virulence, and other cell functions. The majority of discovery of pathogenic *E. coli* sRNA regulation has been done in EHEC. Bhatt et al. (2016) comprehensively reviewed sRNA control in EPEC and EHEC, thus discoveries in this area are mentioned here to outline recent discovery and to highlight their importance in linking metabolism and virulence in EPEC. sRNAs respond rapidly to various environmental changes and are energetically advantageous as regulators because they need not be translated into functional protein to exert regulatory control (Bhatt et al., 2016). sRNAs non-specifically regulate a wide variety of genes with breadth of functions, such as metabolism and virulence genes.

sRNAs are 50 to 500 nt molecules that most often act by post-transcriptionally modulating expression from messenger RNAs (mRNAs) by base-pairing complementary sequences, blocking transcription elongation, translation, and/or mRNA stability (Bhatt et al., 2016). The majority of sRNAs in *E. coli* are *trans-*encoded, or remotely encoded with respect to its target subject, thus create short and often discontinuous complementary segments of 6–25 bp (Bhatt et al., 2016). *Trans-*encoded sRNAs require a chaperone, which is most often Hfq in *E. coli* (Bhatt et al., 2016). The extensive role of Hfq and Hfq-dependent sRNAs in EHEC indicates that there is likely a large role of Hfq-dependent sRNA regulation in EPEC that has not been explored.

Enteropathogenic *Escherichia coli* Hfq-dependent sRNAs MgrR, RyhB, and McaS affect *grlRA* translation (Bhatt et al., 2017) (**Figure 1**). MgrR represses *grlR* directly by binding to the upstream leader region of the mRNA, while activating *grlA* (Bhatt et al., 2017). RyhB directly represses *grlRA* translation by base-pairing to a shorter RNA sequence to repress translation, and McaS likely acts indirectly to repress *grlRA* (Bhatt et al., 2017). These three sRNAs respond to different environmental conditions. Expression of MgrR, which increases expression of the LEE via more stable *grlA* transcripts, increases in the case of low magnesium cation concentrations likely via PhoQ-PhoP phosphorelay (Bhatt et al., 2017). High abundance of iron cations bound to iron regulatory protein Fur repress RyhB, in which case RyhB cannot decrease concentration of the LEE through *grlRA*, thus LEE is activated (Bhatt et al., 2017) (**Figure 1**). The discovery of these three Hfq-dependent sRNAs that specifically regulate EPEC LEE expression is the tip of the iceberg of many sRNA interactions tying environmental sensing and virulence that have yet to be characterized.

Another RNA-binding protein, CsrA (and its homolog RsmA) co-regulates metabolite sensing, motility, stringent response, and virulence via a relaxed sequence specificity that enables base-pairing and regulation of a large number of operons that control many aspects of cell function. CsrA is part of the carbon storage regulation system, which encodes RNAs CsrB and CsrC, as well as protein CsrD that targets these RNAs for RNase E degradation (Bhatt et al., 2009). CsrA is intimately connected to the stringent response, involving DksA and ppGpp. CsrA acts by a number of different mechanisms—some operons are affected when CsrA is in high concentration and creates large homo-multimers, and other operons are affected at lower concentrations (Bhatt et al., 2009). CsrA positively regulates by binding distantly from the Shine–Dalgarno sequence and creating mRNA stability, and negative regulates by binding in proximity to this sequence, which prevents the 30S ribosomal subunit from binding and inhibiting translation (Bhatt et al., 2009). The flagellar operon *flhDC* is likely regulated by CsrA in EPEC because there is a conserved CsrA binding sequence identified in *E. coli* K-12 (Bhatt et al., 2009). It is proposed that CsrA could be involved in differential regulation of that operon in response to glucose concentrations, because the K-12 strain is not motile in 10 mM concentrations of glucose, whereas EPEC is motile (Bhatt et al., 2009).

**FIGURE 2:**
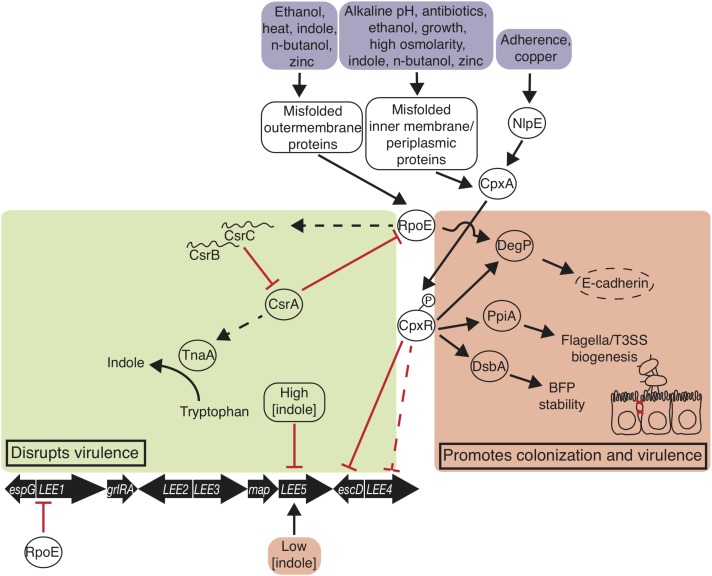
Envelope stress responses disrupt and promote EPEC virulence and colonization. The σ^E^ and Cpx responses are triggered when EPEC encounters environmental signals. σ^E^ promotes *degP* transcription, which degrades host E-cadherin disrupting adherens junctions, and represses *LEE1* transcription. σ^E^ disrupts virulence by decreasing *csrA* transcription. Phosphorylated CpxR promotes *degP, ppiA*, and *dsbA* transcription, and represses *perC* transcription. Indole at 1.5 mM represses *LEE5* transcription and at 50 μM promotes *LEE5* transcription. Thin straight arrows indicate transcription promotion; blunt arrows indicate transcription repression; curved arrow indicates enzymatic reaction; bold arrows indicate genetic elements; proteins are in ovals; environmental signals are in boxes.

CsrA regulates virulence by a number of factors, one of which is by increasing the *sepL* transcript, the product of which facilitates secretion of translocators together with SepD (Bhatt et al., 2009). CsrA represses *grlA* at high concentrations of the regulator, which has downstream negative regulatory effect on LEE expression, possibly in an advantageous effect of conserving resources when glucose is abundant (Bhatt et al., 2009). Importantly, an EPEC strain with *csrA* deleted is unable to create pedestals nor depolarize the enterocyte membrane *in vitro*, in part because the mutant no longer encourages tryptophan metabolism via *tnaA* (Bhatt et al., 2009). CsrA indirectly promotes transcription of *tnaA* (**Figure 2**), which encodes the enzyme tryptophanase of the tri-cistronic operon *tnaCAB* that is similarly tied to both metabolism and virulence (Bhatt et al., 2009). Additionally, CesT protein binds to CsrA, antagonizing the global regulator (Katsowich et al., 2017; Ye et al., 2018). Wide genomic control via sRNAs and their chaperone proteins is coming to light as determinately important in tying environmental stimulation to metabolism and virulence control in strain-specific manners.

## Tryptophan Metabolism and Virulence

The presence of tryptophan has a positive effect on virulence expression in EPEC (**Figure 2**). Overall, tryptophan and *tnaCAB* expression causes an increase in expression of *LEE1, LEE4*, and secreted factors that affect *Caenorhabditis elegans* survival (Bhatt et al., 2011). The *tnaCAB* operon, which encodes a *cis-*acting regulator (TnaC), the catalytic enzyme tryptophanase that cleaves tryptophan into indole, pyruvate, and ammonia (TnaA), and the primary permease important for tryptophan uptake into the bacterium (TnaB), is essential for EPEC-mediated killing of *C. elegans* (Bhatt et al., 2011). At low concentrations, indole promotes *tir* transcription. However, at high concentrations, indole acts as a competitive inhibitor of tryptophanase by binding to the catalytic site which retards LEE transcription (Bhatt et al., 2011) (**Figure 2**). This direct tie between a metabolic gene function and virulence expression is an indication that these cellular processes are delicately intertwined. The exploitation of these connections has already begun in the role of indole and indole-derivatives in suppressing pathogenesis (Bommarius et al., 2013).

Indole and the two indole-derivatives, indole-3-carboxyaldehyde (ICA) and indole-3-acetic acid (IAA) are potential therapeutics because of their repressive effect on LEE expression (Bommarius et al., 2013). Indole is a stationary phase quorum-sensing and signaling molecule produced not only by EPEC, but by resident, non-pathogenic *E. coli*, which potentially explains part of the contribution of resident intestinal microbiota in protecting against pathogenic infection (Bommarius et al., 2013). At the low concentration of 50 μM, indole and ICA induce expression of *tir*, but at higher than 1 mM levels, indole, ICA, and IAA each inhibit expression of LEE genes (Bommarius et al., 2013). ICA most effectively represses LEE expression—at 1.5 mM, ICA reduces pedestals formed by EPEC on cultured cells by sevenfold (Bommarius et al., 2013). When *Citrobacter rodentium* is administered to MyD88^-/-^ mice, a model for EPEC infection in humans, in a lethal dose, oral administration of ICA reduces the CFU in the colon 50-fold (Bommarius et al., 2013). Indole and ICA could be promising therapeutics against EPEC.

## Envelope Stress Responses

Envelope stress responses are linked to virulence attenuation in EPEC (**Figure 2**). Of many stress responses, the alternative sigma factor RpoE (σ^E^) stress response and the two-component system CpxRA are deployed in response to a number of stresses as well as misfolded inner and outer membrane proteins and periplasmic proteins. σ^E^ is one of six alternative sigma factors in *E. coli* (Gruber and Gross, 2003), although the protypical laboratory strain EPEC E2348/69 houses a deleterious mutation in σ^S^ (Bleibtreu et al., 2014). In the σ^E^ stress response, heat (Erickson and Gross, 1989), n-butanol (Rutherford et al., 2010), zinc (Yamamoto and Ishihama, 2005), indole (Yitzhaki et al., 2012), and other factors cause outer membrane protein misfolding that leads to a proteolytic cascade that ultimately shears anti-σ factor RseA, releasing σ^E^ (Mecsas et al., 1993) (**Figure 2**). Growth-phase dependent activation of σ^E^ is ppGpp-dependent (Costanzo and Ades, 2006), and DksA and ppGpp directly activate σ^E^-dependent transcription (Costanzo et al., 2008). σ^E^ associates with RNA polymerase and guides it to promoters to drive expression of chaperone, protease, and outer membrane biogenesis factors (Raivio et al., 2013). CsrA represses σ^E^ translation, and σ^E^ indirectly activates transcription of *csrB* and *csrC*, which bind and sequester CsrA, with potential effects on virulence as stated previously (Yakhnin et al., 2017) (**Figure 2**).

In *E. coli*, the two-component system CpxRA is stimulated by a number of signals, including indole (Raffa and Raivio, 2002), high osmolarity (Jubelin et al., 2005), ethanol (Bury-Moné et al., 2009), n-butanol (Rutherford et al., 2010), copper (Yamamoto and Ishihama, 2006), alkaline pH (Danese and Silhavy, 1998), amino-glycoside antibiotics (Kashyap et al., 2011), adhesion (Otto and Silhavy, 2002), growth (DiGiuseppe and Silhavy, 2003; Wolfe et al., 2008), and a number of membrane/periplasmic protein and phospholipid disruptions (Jones et al., 1997; Wang et al., 2010; Itou et al., 2012; Raivio et al., 2013). Zinc does not induce CpxRA induction (Yamamoto and Ishihama, 2005). Outer membrane lipoprotein NlpE activates CpxA in response to abiotic surface adherence (Otto and Silhavy, 2002), and *cpxP* transcription is transcribed at high levels in the response and blocks phosphorylation of CpxA in a negative feedback loop (Raivio et al., 1999). Inner transmembrane sensory histidine kinase CpxA directly senses stressors, autophosphorylates its cytoplasmic sensory domain, then phosphorylates CpxR, which goes on to bind DNA sequences to regulate gene expression (Grabowicz and Silhavy, 2017) (**Figure 2**). The activation of the Cpx response regulates the LEE and other key proteins that promote bacterial colonization.

Key Cpx response proteins DsbA, PpiA, and DegP are activated by the Cpx response and promote virulence regulation. DsbA is a disulfide oxidase that facilitates stability of the major BFP subunit bundlin (Zhang and Donnenberg, 1996; Heras et al., 2009; Vogt et al., 2010). PpiA is a peptidyl-prolyl-isomerase and may facilitate biogenesis of the type three secretion system and flagella (Justice et al., 2005; MacRitchie et al., 2012). As well as in response to Cpx, protease/chaperone-encoding *degP* is transcribed in response to σ^E^ activation (MacRitchie et al., 2012; Abfalter et al., 2016). DegP degrades misfolded periplasmic envelope proteins and cleaves human E-cadherin, disrupting cell-cell adherens junctions (MacRitchie et al., 2012; Abfalter et al., 2016). α-bundalin of BFP binds to *N*-acetyllactosamine glycan receptors on human enterocytes, and BFP is consequently degraded by DegP (Hyland et al., 2008; Humphries et al., 2010). In addition to these auxiliary factors that contribute to virulence, Cpx has been implicated in direct repression of the LEE.

The Cpx envelope stress response has a negative effect on LEE expression, especially on operons *LEE4* and *LEE5* (MacRitchie et al., 2008) (**Figure 2**). A CpxR binding site at *LEE5* suggests that CpxR may be acting directly at that operon (MacRitchie et al., 2008; Vogt et al., 2010). BFP, Intimin, Tir, and translocator proteins EspA, EspB, EspD, and the needle complex EscF and inner and outer membrane components EscJ and EscC are diminished as consequence (MacRitchie et al., 2008; Vogt et al., 2010; MacRitchie et al., 2012). Despite the positive effect on pathogenesis through BFP and flagella promotion and E-cadherin degradation by some Cpx response proteins (**Figure 2**), the overwhelming effect of envelope stress is a decrease in virulence of EPEC, demonstrated by zinc administration.

Heavy metals induce the envelope stress responses and while most are not feasible as therapies because they are toxic, the World Health Organization has been supplementing with zinc to aid in treating acute diarrheal infections since 2008. Zinc is administered along with rehydration methods to help treat acute diarrheal infections because it has been observed to diminish the severity and duration of disease (Sazawal et al., 1995). Specifically, zinc causes cell envelope stress and indirectly suppresses virulence, possibly through multiple stress mechanisms. For instance, zinc increases transcription of *rpoE* (Xue et al., 2015). Ultimately, there is an observable decrease in expression of *perC*, *bfpA, ler*, and *espA* likely due to indirect downregulation which results in decreased adherence to epithelial cells (Crane et al., 2007; Xue et al., 2015). Additionally, EPEC growth rates decrease and membrane perturbation and permeability increases (Xue et al., 2015). Zinc as a virulence inhibitor has been and continues to be deployed in response to EPEC and other enteric pathogens.

## Conclusion

The study of EPEC virulence has identified the T3SS, many regulators, effector molecules, and environmental stresses that are tied to sensory responses and metabolism, all necessary for pathogenesis. Here we present our current, overall understanding of the complex regulatory networks that promote colonization and virulence, and also those that disrupt this process. Beyond commonly administered antibiotics, the disruption of tryptophan metabolism with indole derivatives, or the now commonly given zinc dietary supplements are two alternate therapy approaches. While ICA and IAA are yet to be used clinically, inexpensive zinc supplements have been given with oral rehydration therapy to children and infants with diarrhea for nearly a decade. Such targeted therapies require an understanding of conserved regulatory pathways. While the majority of studies have been conducted with archetypal EPEC strains, strain-specific regulatory control of virulence genes in response to environmental inputs, like those observed by Hazen et al. (2015a), illustrate the importance of expanding our investigations to encompass a wider variety of related strains (Platenkamp and Mellies, 2017). Research on virulence gene regulation as it relates to environmental cues, stress responses, metabolism, and host-associated signaling will add to the growing body of knowledge necessary for minimizing disease caused by EPEC and related pathogens.

## Author Contributions

JM and AP co-authored and edited the manuscript. AP drew the figures, and compiled the table.

## Conflict of Interest Statement

The authors declare that the research was conducted in the absence of any commercial or financial relationships that could be construed as a potential conflict of interest.
